# Gender and geographic representation in editorial boards of allergy and immunology journals

**DOI:** 10.1186/s13223-026-01034-0

**Published:** 2026-05-04

**Authors:** Ranya Al Jumaily, Natalie DeGurse, M. Elise Graham, Samira Jeimy

**Affiliations:** 1 Department of Clinical Immunology and Allergy, McGill University, Montreal, QC Canada; 2 Department of Internal Medicine, Western University, London, ON Canada; 3 Department of Otolaryngology-Head and Neck Surgery, Dalhousie School of Medicine, Halifax, NS Canada; 4 Department of Clinical Immunology and Allergy, Department of Medicine, Western University, London, ON Canada

**Keywords:** Gender, Allergy, Immunology, Women in medicine, Publications, Research

## Abstract

**Background:**

Editorial boards shape academic publishing, yet disparities in gender and geographic representation persist across disciplines. To date, these disparities have not been comprehensively investigated in Allergy and Immunology journals.

**Objective:**

To examine gender and geographic representation among editorial board members of Allergy and Immunology journals, highlighting disparities and identifying areas for improvement.

**Methods:**

Allergy and Immunology journals were identified using the Clarivate Journal Citation Reports database. Editorial board member data, including names, roles, and locations, were collected from journal websites. Predicted gender was determined using the genderize.io tool. Pearson correlations assessed relationships between journal metrics and editorial board characteristics.

**Results:**

A total of 36 journals comprising 1,546 editorial board members were analyzed. Women accounted for 515/1,546 (33.3%) of all editorial board members and were underrepresented in leadership roles. Specifically, only 11/47 (23.4%) of editors-in-chief and 90/244 (36.9%) of deputy/associate editors were women. Geographically, 1,318/1,546 (85.3%) of editorial board members were based in developed countries, with the United States contributing the highest proportion (348/1,546, 22.5%). Women editors constituted 423/1,318 (32.1%) of board members in developed countries and 92/228 (40.4%) in developing countries. Representation of non-binary and transgender identities could not be reliably determined using the available data.

**Conclusions:**

Gender and geographic disparities are prevalent in Allergy and Immunology journal editorial boards, with significant underrepresentation of women, particularly in leadership roles, and a concentration of editors in developed countries. Addressing these disparities is essential to fostering inclusivity and equity in academic publishing.

## Background

Editorial boards are fundamental to academic publishing, shaping scope and priorities, guiding research dissemination, and defining scholarly discourse. Accordingly, the composition of editorial leadership influences which questions are prioritized and whose perspectives are amplified. Inequities in board composition both mirror and perpetuate systemic biases, with implications for equity, diversity, and inclusion, and may impede the equitable advancement of knowledge within research communities [1].

Although the representation of women in medicine and biomedical research has increased over recent decades, gender disparities in senior academic rank and leadership positions remain substantial. Women are less likely to attain full professorships or senior leadership roles even after adjusting for research productivity and years of experience, indicating persistent structural inequities within academic medicine and related research fields [2–4]. A 2011 audit reported that only 16% of editors-in-chief (EiCs) were women [5]. In 2021, women accounted for 24.4% of EiCs and 27.9% of editorial board members across major medical journals [6–8], demonstrating progress over time. Despite this improvement, women continue to hold roughly one in five editor-in-chief (EiC) positions at leading medical journals, with wide inter-specialty variation as per recent cross-sectional analyses [6–8]. Likewise, investigators from low- and middle-income countries account for only ~ 5% of editorial positions at leading journals, an underrepresentation that may constrain global perspectives and inadvertently deprioritize research and disease burdens affecting developing countries [1].

Despite growing awareness of these disparities within major medical journals, the editorial boards of Allergy and Immunology journals remain underexamined. This gap is notable given the field’s global relevance, encompassing the worldwide burden of allergic diseases and substantial variation in regional healthcare needs and research priorities [9]. Ensuring diverse representation in editorial leadership is critical for fostering equitable academic discourse and driving innovation tailored to diverse populations. Accordingly, this study aims to systematically evaluate gender and geographic representation among editorial board members of Allergy and Immunology journals, identify disparities, and propose actionable strategies for enhancing equity in academic publishing.

## Methods

### Study design

This cross-sectional study evaluated the composition of editorial boards of Allergy and Immunology journals listed in the Clarivate Journal Citation Reports (JCR) database. The analysis was conducted with data between January 1, 2022, and December 31, 2022, to ensure a comprehensive and contemporary dataset.

### Data collection

Data were systematically collected from the official websites of journals identified through the JCR database. Information was extracted for all editorial board members, including their names, roles, geographic location, and subsequently their predicted gender. To ensure the accuracy of the extracted information, multiple reviewers independently verified the data, and discrepancies were resolved through consensus.

Gender was predicted using Genderize.io, an open-access name-to-gender tool that estimates the likelihood that a first name is associated with a male or female gender [10]. The algorithm is trained on large aggregated datasets of first names and their frequency distributions and provides a predicted gender, a probability score (0–1) reflecting confidence in classification, and an observation count. This approach has been used in prior bibliometric studies examining gender representation in academic authorship, including analyses of gender disparities in leading medical journals [11].

We applied a pre-specified probability threshold of ≥ 0.90 to identify high-certainty classifications in the primary analysis. This higher threshold was selected to prioritize classification accuracy while allowing for additional verification of uncertain cases. Names with probability < 0.90 were flagged for manual review. Two investigators independently reviewed publicly available institutional profiles, professional biographies, and other online sources to verify gender classification where possible. When gender could be reasonably inferred through manual verification, the classification was retained. When verification was not possible, the original probabilistic classification provided by Genderize.io was retained to minimize exclusion bias.

To assess the robustness of our findings, a sensitivity analysis excluding names with probability < 0.80 was conducted, thereby restricting the analysis to higher-confidence probabilistic classifications without reliance on manual adjustment.

Although we use the term “gender” throughout this manuscript, we acknowledge the inherent limitations of inferring gender based solely on name-based classification methods. These approaches cannot fully or accurately reflect individuals’ self-identified gender. Gender classification was therefore limited to binary categories due to the constraints of name-based inference tools, and representation of non-binary or transgender identities could not be reliably determined using publicly available information.

1. Names.
2. Roles (e.g., Editor-in-Chief, Deputy/Associate Editor, Other Editorial Board Member).
3. Geographic locations (determined based on institutional affiliations).
4. Predicted gender (determined using the genderize.io database tool, which estimates gender based on names and probability scores).
5. Journal Impact Factor in 2022.

### Data analysis

Descriptive statistics were calculated to summarize gender and geographic distributions across editorial board members. Gender representation was analyzed across editorial roles, including Editors-in-Chief, deputy/associate editors, and other editorial board members. Geographic representation was categorized as either developed or developing countries according to the World Bank 2023 income classification. Pearson correlations were used to explore potential relationships between journal impact factors and editorial board characteristics. Statistical analysis was conducted using numiqo. Statistical significance was defined as *p* < 0.05 [12]. Findings were interpreted within the context of existing disparities in academic publishing, with an emphasis on identifying systemic inequities.

## Results

A total of 36 journals were analyzed, comprising 1,546 editorial board members. Majority was defined as greater than or equal to 55% of board members. Among the editorial boards examined, 30/36 (83.3%) had a majority male board, 3/36 (8.3%) had a majority women board, and 3/36 (8.3%) achieved gender parity (45–55% women).

**Fig. 1:**
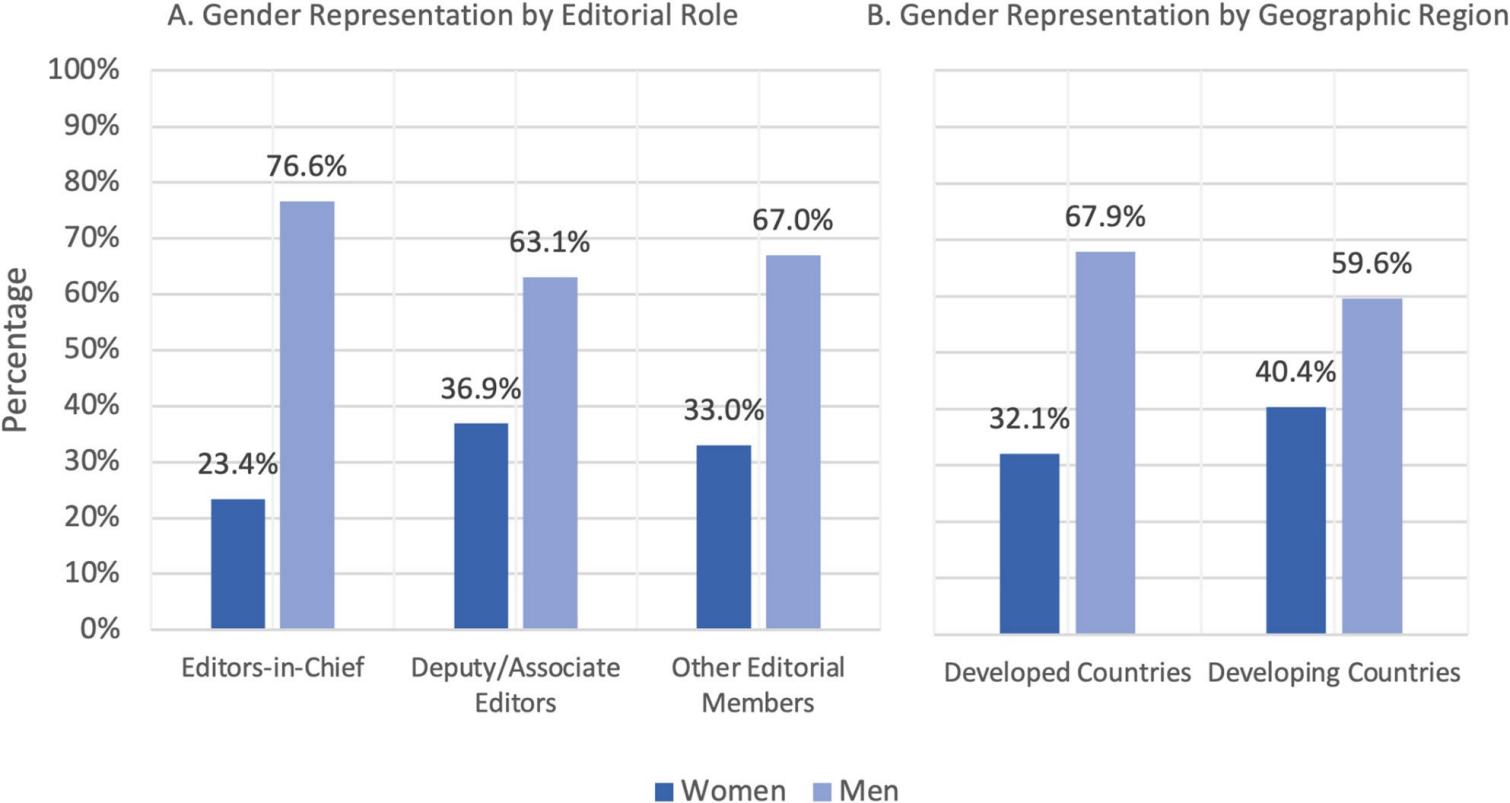
Gender representation across editorial roles and geographic regions among Allergy and Immunology journal editorial boards (*N* = 1,546). **A** Distribution of women and men by editorial role (Editors-in-Chief, Deputy/Associate Editors, and Other Editorial Board Members). **B** Distribution of women and men by geographic region (developed and developing countries)

Overall, women accounted for 515/1,546 (33.3%) of all editorial board members, with substantial underrepresentation in leadership roles. Women comprised 11/47 (23.4%) of Editors-in-Chief (EiC), 90/244 (36.9%) of Deputy/Associate Editors, and 414/1,255 (33.0%) of other editorial board members (Fig. 1). Representation of non-binary or transgender identities could not be reliably determined using available data.

Geographic analysis revealed that 1,318/1,546 (85.3%) of editorial board members were based in developed countries, while 228/1,546 (14.7%) were based in developing countries, according to World Bank 2023 income classification. The United States contributed 348/1,546 (22.5%) of editorial board members. Other major contributors included Poland (136/1,546, 8.8%), South Korea (102/1,546, 6.6%), Spain (94/1,546, 6.1%), and Germany (91/1,546, 5.9%).

Among developed countries, women constituted 423/1,318 (32.1%) of editorial board members. In developing countries, women comprised 92/228 (40.4%) of editorial board members.

In sensitivity analyses excluding editorial board members with gender prediction probability < 0.80, women comprised 468/1,427 (32.8%) of editorial board members compared to 515/1,546 (33.3%) in the primary analysis. Women represented 10/45 (22.2%) of Editors-in-Chief. Among developed countries, women constituted 380/1,214 (31.3%) of editorial board members, and among developing countries, women comprised 88/213 (41.3%). These findings indicate that exclusion of lower-probability classifications did not materially alter overall estimates.

Notably, no significant correlation was observed between journal impact factors and the proportion of women on the editorial boards across both developed and developing countries (*p* = 0.376).

## Discussion

This study highlights gender and geographic disparities in the editorial boards of Allergy and Immunology journals at the time of analysis. Women remain underrepresented, particularly in leadership roles such as EiC, which is consistent with findings in other biomedical disciplines [4–7]. These composition gaps may limit the diversity of perspectives in editorial decision-making, with downstream effects on topic prioritization, reviewer selection, and publication outcomes. Importantly, no significant association was observed between journal impact factor and the proportion of women editors. This finding suggests that gender disparities are not confined to journals of lower prestige but may reflect broader structural patterns within academic publishing.

Geographic disparities were also evident, with editorial board membership heavily skewed toward developed countries. The dominance of editors from the United States and other high-income nations underscores the importance of engaging underrepresented regions to ensure global relevance in research priorities and findings. Encouragingly, women were proportionally better represented on boards in developing countries than in developed countries; this warrants further study to identify contextual factors (e.g., selection pathways, society policies, mentorship structures) that may support gender equity.

Representation of non-binary and transgender identities could not be reliably determined using name-based gender classification methods and publicly available information. This methodological limitation highlights the importance of more inclusive data collection practices in academic publishing to better understand the full spectrum of gender diversity within editorial leadership. Journals should consider strategies to improve representation, including transparent selection processes, mentorship programs for underrepresented groups, and institutional accountability measures.

### Implications for academic publishing

**Fig. 2:**
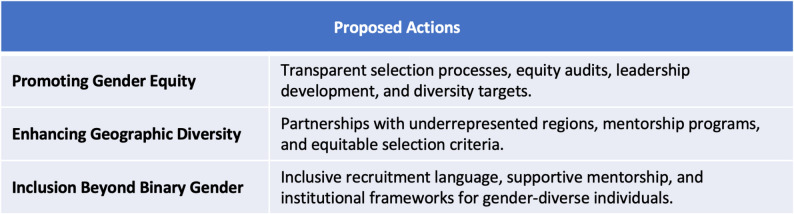
Proposed strategies to enhance equity and diversity in Allergy and Immunology journal editorial membership. Recommendations focus on promoting gender equity, strengthening geographic diversity, and fostering inclusion beyond binary gender frameworks

Addressing these disparities requires concerted efforts by publishers, academic institutions, and funding bodies. Recommendations include (Fig. 2):

1. *Promoting Gender Equity*: Establishing policies to ensure gender balance in editorial leadership roles is essential. This can be achieved through initiatives such as setting clear diversity targets for editorial appointments, implementing gender equity audits within editorial boards, and creating leadership development programs specifically designed to support women in academia. Greater transparency in nomination and selection processes may further promote accountability and inclusivity.
2. *Enhancing Geographic Diversity*: Prioritizing the inclusion of editors from underrepresented regions, particularly in low- and middle-income countries. Specific strategies to achieve this could include fostering partnerships with academic institutions and research organizations in these regions. Journals might also develop editorial training and mentorship programs tailored to researchers from underrepresented areas, providing them with the tools and support to succeed in editorial roles. Examples could include visiting editorships, reducing structural barriers via language-editing support, and travel or meeting stipends. Additionally, establishing transparent and equitable selection criteria can ensure a broader and more inclusive pool of candidates for editorial board positions.
3. *Inclusion Beyond Binary Gender*: Adopting inclusive frameworks to recognize and support gender diversity in academic leadership. Although representation of non-binary and transgender identities could not be reliably assessed in this study due to methodological limitations, journals may consider implementing inclusive recruitment language, mentorship programs for underrepresented gender identities, and institutional mechanisms to better understand and address structural barriers within editorial systems.

## Conclusion

This study underscores the need for continued efforts to address gender and geographic disparities in the editorial boards of Allergy and Immunology journals. Advancing equity may contribute to editorial leadership that better reflects the global research community and enhances the quality and relevance of published literature.

## Data Availability

The data supporting the findings of this study are not publicly available but may be obtained from the corresponding author upon reasonable request.

## Abbreviations

EiC: Editor-in-chief
JCR: Clarivate Journal Citation Reports
